# Myeloid Deletion of Nemo Causes Osteopetrosis in Mice Owing to Upregulation of Transcriptional Repressors

**DOI:** 10.1038/srep29896

**Published:** 2016-07-20

**Authors:** Gaurav Swarnkar, Kyuhwan Shim, Amjad M. Nasir, Kuljeet Seehra, Hung-Po (Tim) Chen, Gabriel Mbalaviele, Yousef Abu-Amer

**Affiliations:** 1 Department of Orthopaedic Surgery, Washington University School of Medicine, 660 South Euclid Avenue, St Louis, MO 63110, USA; 2 Division of Bone and Mineral Diseases, Washington University School of Medicine, 660 South Euclid Avenue, St Louis, MO 63110, USA

## Abstract

The transcription factor NF-κB is central to numerous physiologic processes including bone development, and its activation is controlled by IKKγ (also called NEMO), the regulatory subunit of IKK complex. *NEMO* is X-linked, and mutations in this gene result in Incontinentia Pigmenti in human hemizygous females. In mice, global deficiency causes embryonic lethality. In addition, certain point mutations in the *NEMO* (*IKBKG)* human gene manifest skeletal defects implicating NEMO in the regulation of bone homeostasis. To specifically investigate such role, we conditionally deleted *Nemo* from osteoclast and myeloid progenitors. Morphometric, histologic, and molecular analyses demonstrate that myeloid NEMO deletion causes osteopetrosis in mice. Mechanistically, NEMO deficiency hampered activation of IKK complex in osteoclast precursors, causing arrest of osteoclastogenesis and apoptosis. Interestingly, inhibiting apoptosis by genetic ablation of TNFr1 significantly increased cell survival, but failed to rescue osteoclastogenesis or reverse osteopetrosis. Based on this observation, we analyzed the expression of different regulators of osteoclastogenesis and discovered that NEMO deletion leads to increased RBPJ expression, resulting in a decrease of Blimp1 expression. Consequently, expression of IRF8 and Bcl6 which are targets of Blimp1 and potent osteoclastogenic transcriptional repressors, is increased. Thus, NEMO governs survival and osteoclast differentiation programs through serial regulation of multiple transcription factors.

The transcription factor NF-κB, which encompasses a family of transcription factors, was initially identified in B cells and subsequently was shown to be ubiquitously expressed in all cell types^1^. Although early studies highlighted the role of NF-κB as a modulator of innate and adaptive immunity, subsequent research established a central role for this factor in ample physiologic cellular functions^2^. More importantly, NF-κB was further implicated in pathological conditions and diseases, including inflammation, cancer, metabolic disorders, and skeletal disparities, prompting the design of NF-κB based relevant therapeutic modalities^3^,^4^,^5^.

NF-κB signaling entails transmission of receptor-activating signals leading to assembly and activation of a signalosome complex that includes adaptor proteins and kinases. The most notable members of this complex are TRAF protein ligases, the scaffold protein IKKγ/NEMO and the catalytic subunits IKK1 and IKK2. Signal-specific recruitment, assembly and activation of this complex lead to further recruitment of substrates such as the NF-κB inhibitory protein IκB. Once at the proximity of IKK, IκB undergoes phosphorylation, ubiquitination and proteosome-mediated degradation. Subsequently, the active subunits of NF-κB including p65/RelA, p50 are released, translocate to the nucleus, bind to consensus DNA motifs, and initiate transcription^6^,^7^,^8^,^9^,^10^.

The individual role of most members in the NF-κB signaling in various cells has been studied in details using gene knockout studies^6^,^11^,^12^. The surprising discovery in the late 1990s that NF-κB is key regulator of bone homeostasis^13^ ignited a new era of discoveries aimed at understanding the role of NF-κB members and signals in bone health and disease. In this regard, tissue-specific deletion of most members of the NF-κB pathway, notably IKK1, IKK2, IκB, RelA, and combined deletion of p50/p52 displayed skeletal anomalies^3^,^14^,^15^. However, the direct role of NEMO in bone homeostasis was not described. Nevertheless, numerous studies suggest that NEMO is indeed an essential component of NF-κB mediated skeletal homeostasis. In this regard, we and others have shown that NEMO binding domain peptide, which acts as a decoy to attenuate IKK activation, inhibits osteoclasts and osteolysis^16^,^17^,^18^,^19^,^20^,^21^. Furthermore, several patient case reports described association between specific NEMO mutations and abnormal skeletal manifestations^22^,^23^,^24^,^25^. These observations suggest that NEMO maybe directly required for skeletal development and homeostasis.

Despite this massive effort, decoding the precise repertoire of NF-κB action under physiologic and pathologic conditions remains an overwhelming challenge. In this regard, a relentless effort continues to decipher the molecular role of IKKγ/NEMO as a signal integrator and a scaffold protein pairing upstream cellular responses with kinase-targeted selection of appropriate substrates in a cell-specific manner^26^. Supporting this contention is a recent report indicating that NEMO determined stimulus-specific transduction by directing the IKK complex hub toward IκBα^27^. Thus, it appears that the extent of NEMO functions as a master regulator of NF-κB signaling remains underappreciated.

In order to examine the role of NEMO in skeletal development, we conditionally deleted the gene in the myeloid compartment using various monocyte/macrophage and osteoclast lineage-specific Cre deleters. Mice devoid of NEMO in these cells developed severe osteopetrosis owing to defective osteoclastogenesis. At the molecular level, this defect arises from dysregulation of NF-κB-dependent transcriptional machinery in the myeloid lineage.

## Results

### Mice lacking NEMO in myeloid lineage are smaller and display developmental disparities

**Figure 1:**
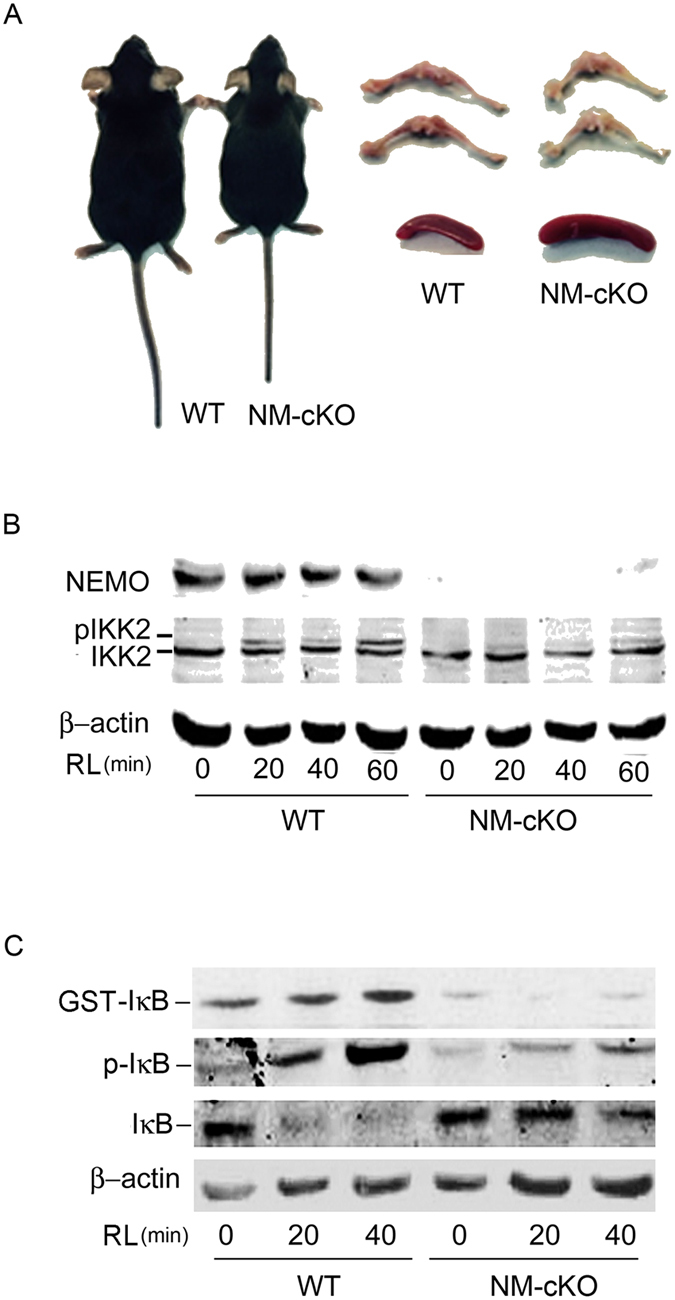
NEMO-myeloid deficient mice are smaller and display developmental defects. NEMO was deleted using LysM and CTSK mediated cre. (**A**) Photomicrograph of Lysozyme-cre-NEMO floxed (NM-cKO^−LysM^) mice (left panel) and bone and spleen (right panel) compared to WT mice. (**B**,**C**) Western blot analysis of WT and NM-cKO cells lysates showing deletion of NEMO (48 kDa, panel B) and attenuation of NF-κB signaling as evident by lack of IKK2 (p-IKK2 = 87 kDa, IKK2 = 85 kDa) and IκB (p-IκB = 40 kDa, IκB = 39 kDa) phosphorylation in panels B and C, respectively, in NEMO null cells upon RANKL stimulation (50 ng/mL) for the time points indicated. β-Actin (42 kDa) was used as loading control.

To assess the direct role of NEMO in skeletal development, we specifically deleted NEMO in myeloid progenitor cells and osteoclast-committed precursor cells by crossing *Nemo*-floxed mice with Lysozyme-M-Cre (NM-cKO^−LysM^, referred to as NM-cKO throughout the manuscript) or Cathepsin-K (CTSK)-Cre (NM-cKO^−CTSK^), respectively, at early (progenitor) or late (osteoclast) lineage differentiation stages. Initial examination shows that NM-cKO mice are smaller in size and display growth and developmental retardation (Fig. 1A left panel). Further examination shows that long bones of NM-cKO are hypo-cellular and these mice displayed splenomegaly consistent with extra-medullary hematopoiesis (Fig. 1A right panel). Genotyping PCR (not shown) and Western blot analyses confirmed efficient deletion of NEMO evident by minimal expression of NEMO in NM-cKO mice (Fig. 1B, top panel). This was further supported by failure to activate IKK2 (Fig. 1B, middle panel) and subsequent phosphorylation of IκB (p-IκB) or phosphorylation of exogenous GST-IκB substrate (Fig. 1C) following treatments with receptor activator of nuclear factor kappa-B ligand (RANKL or RL), confirming that NEMO is required for RANKL-induced activation of NF-κB pathway.

### Myeloid-specific deletion of NEMO causes an osteopetrotic phenotype in mice

**Figure 2:**
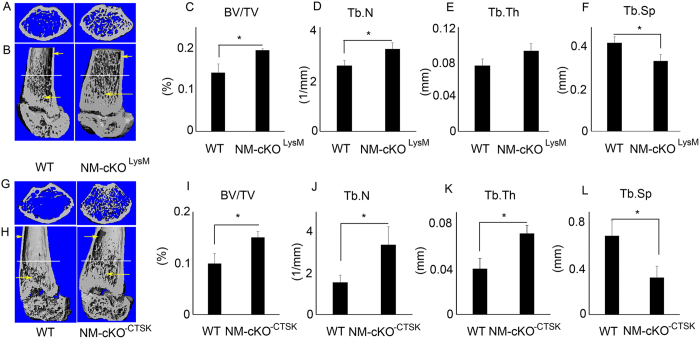
Myeloid-specific deletion of NEMO in mice leads to osteopetrotic bone phenotype. (**A**,**B**) Micro-CT images of distal femur diaphysis and quantification of (**C**) bone volume (BV/TV), (**D**) Trabecular number (Tb.N), (**E**) Trabecular thickness (Tb.Th) and (**F**) Trabecular spacing (Tb.Sp) from female, 5 weeks old control and NM-cKO^LysM^ mice. Lower panel; (**G**,**H**) Micro-CT images and quantification of (**I**) bone volume (BV/TV), (**J**) Trabecular number (Tb.N), (**K**) Trabecular thickness (Tb.Th) and (**L**) Trabecular spacing (Tb.Sp) from female, 5 weeks control and NM-cKO^−CTSK^ mice (n = 6–8; *P < 0.05). Arrows point to cortical and trabecular regions. Horizontal lines across the diaphysis in B and H indicate approximate locations from which images A and G, respectively, were constructed.

Next, we examined the effect of NEMO deletion on skeletal development. Micro-CT analysis of the long bones indicates that both NM-cKO^−LysM^ (Fig. 2A–F) and NM-cKO^−CTSK^ (Fig. 2G–L) mice show denser bones in both the trabecular (Fig. 2A,G) and cortical regions (Fig. 2B,H; arrows). Consistently, all bone parameters including BV/TV (Fig. 2C,I), trabecular number (Fig. 2D,J), trabecular thickness (Fig. 2E,K) and trabecular spacing (Fig. 2F,L) indicate that both genotypes of NM-cKO mice display osteopetrotic phenotype. These results confirm that NEMO is critical for normal skeletal development at early and late stages.

### Osteopetrosis in NEMO-null mice is due to hampered osteoclast differentiation and decreased cell survival

**Figure 3:**
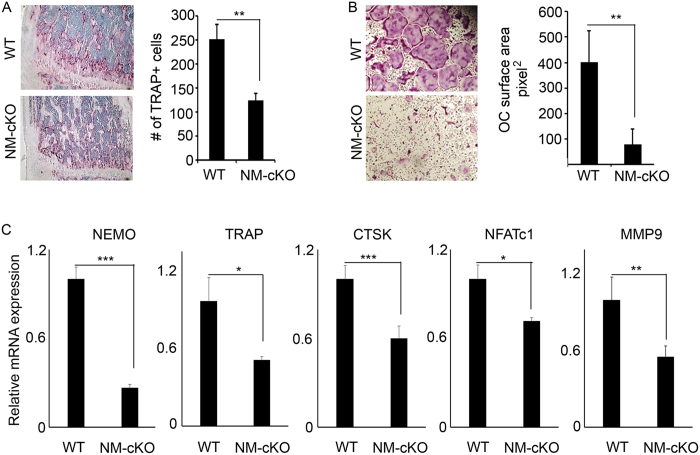
Osteopetrosis in NEMO-null mice is due to hindered osteoclast differentiation. (**A**) WT and NM-cKO (NM-cKO^−LysM^ ) mice (3-4 weeks old; n = 6 per group) were sacrificed, and long bones were processed for histology, stained with TRAP (left panel) to visualize osteoclasts and innumerate osteoclast counts per bone area (right panel) using Osteomeasure. (**B**) BMMs were isolated from NM-cKO and WT mice. Cells were plated with M-CSF (20 ng/mL) and RANKL (50 ng/mL) for 4 days and then fixed and TRAP-stained (left panel) and counted (right panel). (**C**) PCR quantification of osteoclast genes *ikbkg* (*NEMO*), *tracp* (TRAP), Cathepsin-K (*CTSK*), NFATc1, and MMP9. (*P < 0.05, **P < 0.01, ***P < 0.001).

To better understand the mechanism underlying the osteopetrotic phenotype of NM-cKO mice, we examined tartrate-resistant acid phosphatase (TRAP)-stained histological sections of the trabecular regions of long bones and found significantly less osteoclasts in NM-cKO compared with wild type bone sections (Fig. 3A). This finding suggested that impaired osteoclastogenesis *in vivo* is likely the primary culprit of osteopetrosis in NM-cKO mice. To further decipher the details of this osteoclast abnormality, we examined differentiation and survival of NEMO-deficient progenitors and pre-osteoclasts *in vitro*. The results indicate that NM-cKO-derived marrow cells exhibit impaired osteoclast differentiation potential evident by significantly less osteoclast formation *in vitro* (Fig. 3B) and by reduced expression of molecular markers of osteoclast differentiation, including TRAP, Cathepsin-K (CTSK), Matrix metallopeptidase 9 (MMP9), and Nuclear Factor of Activated T-Cells Calcineurin-Dependent 1 (NFATc1) (Fig. 3C).

**Figure 4:**
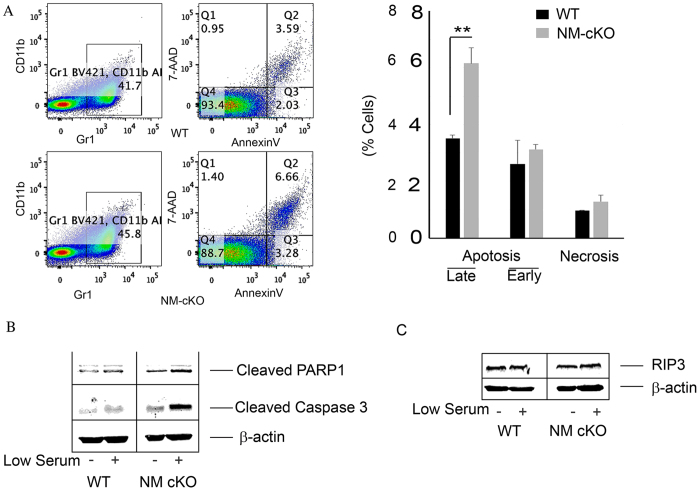
Apoptosis in myeloid-specific NEMO-null mice. (**A**) BMMs were isolated from NM-cKO (NM-cKO^−LysM^) and WT mice, cultured overnight in α-MEM supplemented with 10% FBS and 10 ng/mL M-CSF and stained for CD11ba and Gr1 followed by Annexin V/7AAD staining. FACS analysis was done to determine apoptosis in these cells. The Annexin V positive cells (Q2 - late; Q3- early) in FACS (left panels) and quantitation (right panel) are shown. (**P < 0.01) (**B**) Western blot indicating increased cleaved PARP1 (89 kDa) and cleaved Caspase 3 (17 kDa) levels in NM-cKO cell under basal and serum starved condition when compared to WT cells. (**C**) Western blot indicating no change in RIP3 (60 kDa) expression in NM-cKO cells when compared with WT cells.

The hypo-cellularity of the long bones suggested a potential cell survival impediment. To scrutinize this possibility, we analyzed apoptosis using fluorescence-activated cell sorting (FACS)-based apoptosis and Western blot assays. Indeed, our results confirmed that NM-cKO myeloid progenitor cells are prone to apoptosis, but not necrosis, as shown by increased staining for the apoptotic marker annexin V within the CD11b+/Gr1+ NM-cKO cell populations (Fig. 4A, lower panels) when compared to WT cells (Fig. 4A, top panels). Similarly Western blot data indicates an increase in cleaved Caspase-3 and PARP1 expression (Fig. 4B). Consistent with FACS data, there was no change in RIP3 expression between both phenotypes (Fig. 4C), indicating that necrosis is not the mechanism underlying cell death in *Nemo*-deficient cells.

### Neutralization of TNFα or deletion of TNFr1 protects NEMO- null cells from apoptosis but fails to rescues defective osteoclastogenesis

**Figure 5:**
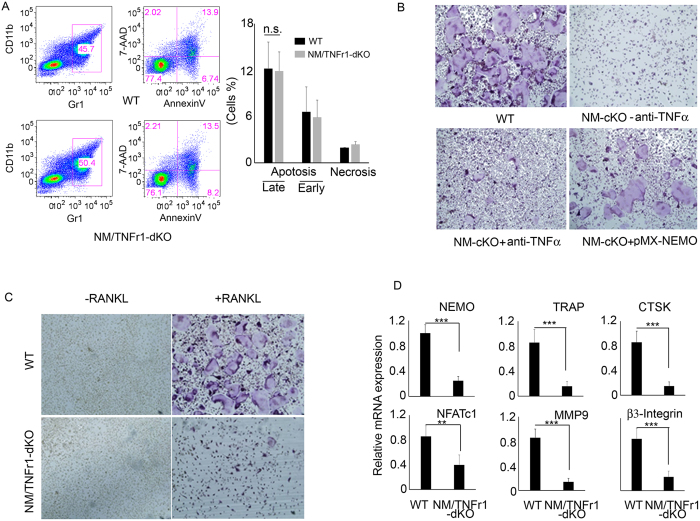
Assessment of apoptosis and osteoclastogenesis after neutralization of TNFα or deletion of TNFr1 in NEMO-null cells. (**A**) FACS analysis of BMMs isolated from NEMO/TNFr1 double KO (NM/TNFr1-dKO) and WT mice and stained for CD11ba and Gr1 followed by Annexin V/7AAD staining. Annexin V positive cells in FACS (left panel) and quantitation (right panel) are shown. (**B**) BMMs were isolated from WT and NM-cKO mice. NM-cKO cells were cultured with either TNFα neutralizing antibody or infected with WT-NEMO retrovirus (pMX-NEMO) for 4 days in the presence of M-CSF (20 ng/mL) and RANKL (50 ng/mL) and then fixed and TRAP-stained. (**C**) BMMs were isolated from NEMO/TNFR1 double KO (NM/TNFr1-dKO) and WT mice and cultured in the presence of M-CSF (20 ng/mL) and RANKL (50 ng/mL) for 4 days and then fixed and TRAP-stained. (**D**) PCR quantification of OC genes NEMO, TRAP, CTSK, NFATc1, β3-integrin, and MMP9 from WT and (NM/TNFr1-dKO) BMMs. (**P < 0.01, ***P < 0.001).

To test if cell apoptosis is the underlying cause of reduced osteoclastogenesis and osteopetrosis in NM-cKO, we employed *in vitro* and *in vivo* approaches. First, NM-cKO-derived progenitor cells were incubated in the continuous presence of TNFα neutralizing antibody to avert apoptosis, and osteoclastogenesis was measured. Second, NM-cKO mice were bred with germline *TNFr1*-null mice to generate NEMO/TNFr1 double knockout mice (NM/TNFr1-dKO) to attenuate any possible apoptotic signal at the outset of NEMO deletion. FACS analysis shows that neutralization of TNFα restored survival and protected NM-cKO cells from apoptosis (Fig. 5A; compare with Fig. 4). This rescue, however, failed to recapitulate osteoclastogenesis (Fig. 5B). In contrast, osteoclastogenesis and cell survival were restored by retroviral expression of WT-NEMO (pMX-NEMO) in the NM-cKO cells (5B) buttressing the requirement of NEMO for cell survival and osteoclast differentiation. Consistent with these results, bone marrow cells from NM/TNFr1-dKO animal failed to differentiate into osteoclasts as evident by TRAP staining (Fig. 5C) and by mRNA expression of osteoclast marker genes (Fig. 5D). In summary, in both *in vitro* and *in vivo* cases, osteoclastogenesis was significantly reduced with persistent skeletal osteopetrotic features in the NM/TNFr1-dKO mice (not shown). Collectively, these data suggest that intact NEMO is essential for NF-κB signaling, myeloid lineage survival and osteoclast differentiation.

### NEMO regulates osteoclastogenesis via RBPJ-Blimp1 axis

**Figure 6:**
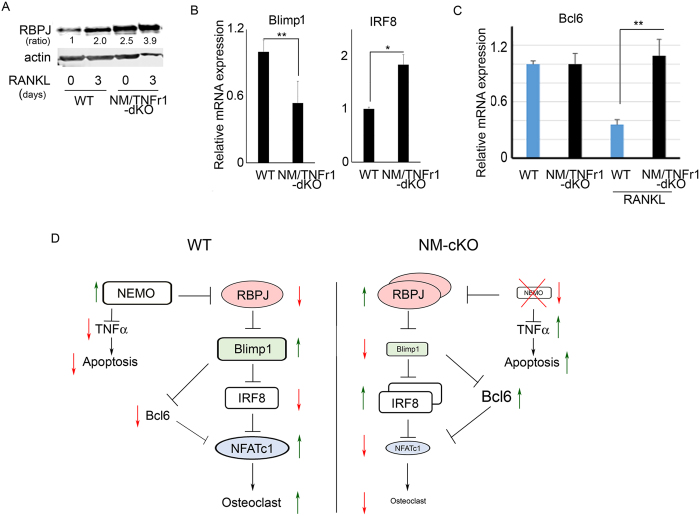
NEMO regulates osteoclastogenesis via inhibiting RBPJ. (**A**) WT and NM/TNFr1-dKO BMMs were treated with RANKL for 0 and 3 days, lysed, and subjected to immunoblotting with RBPJ (56 kDa) antibody (1:1000). (**B**,**C**) Q-PCR quantification of relative mRNA expression (normalized with actin) of Blimp1, IRF8, and Bcl6 in WT and NM/TNFr1-dKO cells under the indicated conditions (+/−RANKL). (*P < 0.05, **P < 0.01). (**D**) Proposed model of NEMO function during osteoclastogenesis. Left and right panels illustrate regulation of osteoclastogenesis under NEMO sufficient or null conditions, respectively.

Despite rescue of the apoptotic phenotype of NM-cKO, NEMO deficient BMMs failed to differentiate into osteoclasts. To better clarify the possible mechanism regulating NEMO function in these cells, we studied the expression of other known regulators of osteoclastogenesis. Recombination signal binding protein for immunoglobulin kappa J region (RBPJ) has been described as a negative regulator of osteoclastogenesis. Specifically, it plays a repressor role during osteoclastogenesis especially under inflammatory conditions. In this respect, we showed recently that inactivation of NF-κB signaling is accompanied with accumulation of RBPJ, which along with other co-repressors inhibit osteoclasts^28^. Furthermore, RBPJ has been shown to regulate osteoclastogenesis via different mechanisms, such as TAK1-RBPJ axis^28^, Blimp1-IRF8 axis^29^ or via regulating TGFβ –PLCγ2 axis^30^. To this end, we measured the expression of RBPJ, Blimp1, IRF8 and Bcl6 in NEMO/TNFr1-dKO cells. Our Western blot results indicate that under NEMO deficiency, RBPJ expression was significantly increased under basal (2.5 fold) and RANKL (3.9 fold) stimulated conditions (Fig. 6A). qPCR analysis confirmed significant decrease in the expression of RBPJ target, B lymphocyte-induced maturation protein-1 (Blimp1), and subsequent increased interferon regulatory factor 8 (IRF8) expression (Fig. 6B), which can inhibit osteoclastogenesis by suppressing expression of the osteoclast master regulator NFATc1 (see Fig. 5D). Under similar culture conditions we found that whereas expression levels of the NFATc1 repressor B cell lymphoma-6 (BCL6) were reduced in RANKL-treated wild type cells to permit osteoclastogenesis, no such reduction was evident in NM/TNFr1-dKO cells when compared to untreated cells (Fig. 6C). Collectively, these data (summarized in Fig. 6D) suggest that during osteoclastogenesis, absence of NEMO attenuates NF-κB signaling which leads to elevated levels of the transcriptional repressor RBPJ. RBPJ in turn inhibits Blimp1 leading to increased expression of IRF8 and Bcl6, both of which inhibit expression of the master osteoclast gene NFATc1 (see also Figs 3C and 5D) and attenuate osteoclastogenesis. In addition, NEMO deletion leads to cell apoptosis through TNFα-mediated caspase signals.

## Discussion

The transcription factor NF-κB is a master regulator of ubiquitous cellular functions and cell fate, and is essential for maintaining physiologic homeostasis^31^. NEMO, which lacks catalytic activity, serves as a scaffold platform to regulate IKK complex assembly^32^. Furthermore, recent studies suggest that NEMO is a signaling hub that facilitates and couples various signals with downstream substrates^27^. Therefore, understanding the intricate details of NEMO-mediated signals will open new and unexplored research opportunities. Although the role of several NF-κB molecules in skeletal homeostasis has been elucidated^12^,^15^, the direct role of NEMO, the master regulator of NF-κB pathways, in bone has not been directly addressed.

In this study, we document two major observations. First, we show that tissue-specific deletion of NEMO in the myeloid lineage compromises cell survival. Second, this deletion leads to the development of skeletal disparities, primarily osteopetrosis. The pro-survival role of NF-κB signaling has been further elucidated and our data ascertain that NEMO plays an essential role in this phenomenon. More importantly, we provide clear evidence that restoring cell survival by attenuating death signals, typically transmitted through TNFr1 signaling, does not restore osteoclastogenesis. Thus, NEMO plays multiple non-redundant roles in cell survival and osteoclastogenesis. We further posit that the osteopetrotic phenotype arises directly from defective osteoclastogenesis and from scarcity of osteoclast progenitor cells in the marrow cavity of long bones, as evident by hypo-cellularity in the tibiae and femora of NM-cKO mice. Intriguingly, we observed splenomegaly in NM-cKO mice, indicating extra-medullary hematopoiesis and obstructive marrow hematopoiesis. Thus, NEMO expression and baseline NF-κB activity are required for normal bone marrow hematopoiesis.

Osteoclastogenesis is governed by intricate transcriptional machinery at the apex of which is NF-κB-mediated basal transcription. Further, ample evidence suggest that NF-κB regulates transcription of numerous genes that determine the fate of osteoclast differentiation and survival^12^. In this regard, we have shown recently that deletion of TGFβ-activated Kinase-1 (TAK1) and subsequent impairment of NF-κB activation is accompanied with high expression of the transcription factor RBPJ, which acts as a transcriptional repressor of osteoclastogenesis^28^. Earlier studies have shown that the NF-κB member p65/RelA, an immediate downstream target of NEMO/IKK2 classical pathway, and RBPJ share an overlapping binding site at different gene promoters, including NFATc1^33^,^34^. In other studies, our group and Zhao *et al*. showed that RBPJ inhibits osteoclastogenesis by inhibiting NFATc1 expression via suppressing Blimp-1, which prevents the downregulation of the transcriptional repressor IRF8^29^,^35^. Furthermore, Miyauchi *et al*. showed that osteoclast transcriptional repressor B cell lymphoma-6 (Bcl6) is a direct target of Blimp1^36^. Our study provides compelling evidence that inactivation of NF-κB through deletion of NEMO facilitates accumulation of RBPJ and associated co-repressors. Our data further show that under these conditions, expression levels of Blimp1, a target of RBPJ and inducer of osteoclastogenesis, are reduced, hence permitting high expression of IRF8 and Bcl6, both of which repress expression of the osteoclast master regulator NFATc1 and impede osteoclastogenesis (Fig. 6D). It is important to note that NEMO appears to be essential for RANKL-induced repression of Bcl6 (Fig. 6C), which subsequently permits osteoclast gene transcription. In this regard, it has been proposed that some macrophage-specific enhancers contain NF-κB binding sites and recruit such factors upon stimulation and that these enhancers are negatively inhibited by sequence-specific transcriptional repressors, namely Bcl6^37^. We posit that NEMO deletion, which plummets NF-κB transcriptional activity, permits RANKL-specific enhancer repression through elevated levels of Bcl6. This is a novel finding that warrants further investigation. Taken together, our study identifies NEMO as a RANKL-dependent regulator of transcriptional machinery that controls osteoclastogenesis and bone homeostasis.

## Methods

### Animals

*Nemo* floxed mice on a C57BL/6 background, generously provided by Dr. Manolis Pasparakis (Cologne, Germany) were crossed with LysM-Cre (Jacksons Laboratories) and CTSK-cre mice^38^ to produce heterozygous mice. The NEMO heterozygous mice were further intercrossed to generate homozygous null mice (NM-cKO^−LysM^ or NM-cKO^−CTSK^). To delete the *TNFr1 gene*, germline TNFr1-null mice (Jacksons Laboratories) were crossed with NM-cKO^−LysM^ to generate heterozygous mice, which were further intercrossed to generate homozygous NEMO and TNFr1 double knock out mice (NM/TNFr1-dKO). Mice were housed at the Washington University School of Medicine barrier facility. All experimental protocols were carried out in accordance with the ethical guidelines approved by the Washington University School of Medicine Institutional Animal Care and Use Committee.

### Cell culture and osteoclastogenesis

Bone marrow cells were cultured in α-MEM supplemented with 100 units/mL penicillin/streptomycin and 10% FBS (v/) with 10 ng/mL M-CSF for 16 h to separate adherent cells from non-adherent cells. Non-adherent cells were harvested and used as enriched bone marrow–derived monocyte/macrophage precursors (i.e., BMMs). BMMs were further cultured with M-CSF (20 ng/mL) and RANKL (50 ng/mL) for 4 days followed by fixation and TRAP staining using TRAP-Leukocyte kit (Sigma, St Louis, MO, USA). TRAP-positive cells containing more than three nuclei were considered multinucleated *bona fide* osteoclasts. In certain conditions, NM-cKO cells were either cultured with TNFα neutralizing antibody (0.2 μg/mL) or transfected with retroviral pMX-WT-NEMO generated by transfecting pMX-WT-NEMO in Plat-E cells as described previously^28^.

### Western blot analysis

To study phosphorylation and expression of proteins in osteoclasts, BMMs were induced with RANKL for different time points as described in the figures and the total cell lysates were prepared in cell lysis buffer (Cell Signaling Technology, Danvers, MA, USA). Protein concentration was determined and equal amount of protein was applied onto SDS-PAGE. After the transfer, membranes were blocked in 5% skimmed milk for 1 h at room temperature, followed by probing with specific primary antibody primary antibodies in 5% BSA in PBS-Tween (1% v/v) overnight and then washed three times with PBS-Tween (PBST) and probed with secondary antibodies from LI-COR (Odyssey Imager; donkey anti-rabbit/IRDye 800 CW/anti-goat/IRDye 800 CW anti-mouse IRDye 680 CW) for 1 h at room temperature. Membranes were then washed three times with PBST and scanned by using LI-COR Odyssey Imager (LI-COR Biosciences, Lincoln, NE, USA). The RBPJ, NEMO, IKK2, pIκB, IκB and cleaved PARP1 antibodies were purchased form Santa Cruz, Dallas, TX, USA; Cleaved Caspase 3 antibody was purchased from Cell Signaling Technology, Danvers, MA, USA; β-actin was purchased from Sigma, St Louis, MO, USA.

### *In vitro* kinase assay

WT and NM-cKO BMMS were treated with RANKL for 0, 20 and 40 minutes followed by lysis in RIPA buffer (50 mM Tris (pH 7.4), 150 mM NaCl, 1% NP-40, 1 mM EDTA 50 μg/ml leupeptin, 10 μg/ml aprotinin, 50 μg/ml phenylmethylsulfonyl fluoride (PMSF), 0.2 mM sodium orthovanadate, 100 mM sodium fluorine) for immunoprecipitation. IKK2 complex was immunoprecipitated with NEMO FL-419 (Santa Cruz, Dallas, TX, USA) antibody, washed twice with IP buffer, once with kinase assay buffer (Cell Signaling Technologies Danvers, MA, USA), and incubated for 30 minutes at 30 °C in 30 μl kinase assay buffer with 1 μg GST-IκBα, 2.5 mM MgCl2, and 16 μM ATP. Reaction was terminated with 10 μl 4 X reducing sample buffer. Samples were analyzed by Western blot.

### Micro-CT acanning and analysis

Intact femur and tibia from WT mice and NM-cKO animals were isolated, fixed overnight in 10% formalin followed by washing with Phosphate Buffer Saline (PBS) 3 times and transferred to 70% ethanol (v/v). Bones were then scanned using Scanco Medical micro-CT systems (Scanco, Wayne, PA, USA) at the core facility at the musculoskeletal center at Washington University (St. Louis, MO). Briefly, Images were scanned at a resolution of 20 μm, slice increment 20 μm, voltage 55 kV, current 145 μA and exposure time of 200 ms. After scanning the whole bone, contour was drawn from the growth plate toward trabecular and cortical regions of tibia of approximately 200 slices were analyzed for WT and NM-cKO bones, and 3D images were constructed.

### Histology

Femur and tibia were collected from mice and fixed in 10% buffered formalin for 16–24 h. Bones were then decalcified for 2 weeks in decalcification buffer (14% (w/v) EDTA, NH_4_OH, pH 7.2), dehydrated in graded ethanol (30–70%), cleared through xylene, and embedded in paraffin. Paraffin sections were stained for TRAP to visualize osteoclasts.

### qPCR analysis

Cells were cultured in presence of M-CSF (20 ng/mL) and RANKL (50 ng/mL) for 3 or 4 days as labeled in the figures. mRNA was isolated using PureLink RNA mini kit (Ambion, Grand Island, NY, USA) and cDNA were prepared using high capacity cDNA reverse transcription kit (Applied Biosystems). qPCR was carried out on BioRad CFX96 real time system using iTaq universal SYBR green super-mix (BioRad, Hercules, CA, USA). mRNA expression were normalized using actin as a housekeeping gene. The following primers were used for qPCR analysis. TRAP-F: CGACCATTGTTAGCCACATACG, TRAP-R: CACATAGCCCACACCGTTCTC, CTSK-F: ATGTGGGTGTTCAAGTTTCTGC, CTSK-R: CCACAAGATTCTGGGGACTC, MMP9-F: ACTGGGCTTAGATCATTCCAGCGT, MMP9-R: ACACCCACATTTGACGTCCAGAGA, NFATc1-F: CCGGGACGCCCATGCAATCTGTTAGT, NFATc1-R: GCGGGTGCCCTGAGAAAGCTACTCTC, NEMO-F: CTAGGAGCTCCGGTTCTGTC, NEMO-R: TGCTTGTTCATCCAACAGGA, IRF8-F: GAGCGAAGTTCCTGAGATGG, IRF8-R: TGGGCTCCTCTTGGTCATAC, Blimp1-F: GACGGGGGTACTTCTGTTCA, Blipm1-R: GGCATTCTTGGGAACTGTGT, Bcl6-F: AGACGCACAGTGACAAACCATACAA, Bcl6-R: GCTCCACAAATGTTACAGCGATAGG.

### FACS analysis

Total bone marrows cells were cultured in α-MEM supplemented with 10% FBS and 10 ng/mL M-CSF for 16 hours. Afterwards, cells were washed twice with cold PBS, re-suspended in FACS buffer (PBS supplemented with 0.5% BSA and 2 mM EDTA), incubated with Fc blocker (BD Biosciences, San Jose, CA, USA) for 15 minutes before staining with APC Alex 780 conjugated anti-CD11b antibody (eBiosciences, San Diego, CA, USA) and Brilliant Violet 421 conjugated Gr-1 antibody (Biolegend, San Diego, CA, USA) for 30 minutes. Antibody-labelled cells were then washed with FACS buffer, resuspended in Annexin V binding buffer and stained with PE conjugated AnnexinV (BD Biosciences, San Jose, CA, UA) for 15 minutes. After adding 7-AAD, flow cytometry data acquisition was performed on FACScan at Washington University High Speed Cell Sorting Core Facility and analyzed by FlowJo Version 10.1 (TreeStar, Ashland, OR, USA).

### Statistical analysis

All experiments were repeated at least three times. Statistical analyses were performed by using Student *t*-test. Multiple treatments were analyzed by using one-way ANOVA followed by post hoc Newman-Keuls test of significance. Values are expressed as mean ± SD of at least three independent experiments. *P* values are indicated where applicable.

## Additional Information

**How to cite this article**: Swarnkar, G. *et al*. Myeloid Deletion of Nemo Causes Osteopetrosis in Mice Owing to Upregulation of Transcriptional Repressors. *Sci. Rep.* **6**, 29896; doi: 10.1038/srep29896 (2016).
